# *Ganoderma lucidum* polysaccharide peptide prevents renal ischemia reperfusion injury via counteracting oxidative stress

**DOI:** 10.1038/srep16910

**Published:** 2015-11-25

**Authors:** Dandan Zhong, Hongkai Wang, Ming Liu, Xuechen Li, Ming Huang, Hong Zhou, Shuqian Lin, Zhibin Lin, Baoxue Yang

**Affiliations:** 1 State Key Laboratory of Natural and Biomimetic Drugs, Department of Pharmacology, School of Basic Medical Sciences, Peking University, Beijing, China; 2 Fuzhou Institute of Green Valley Bio-Pharm Technology, Fuzhou, China; 3 JUNCAO Technology Research Institute, Fujian Agriculture and Forestry University, Fuzhou, China

## Abstract

*Ganoderma lucidum* polysaccharide peptide (GLPP) scavenges oxygen free radicals that are a key factor in the pathogenesis of renal ischemia reperfusion injury (RIRI). The aim of this study was to determine whether GLPP could attenuate RIRI by counteracting the oxidative stress. The mechanism involved was assessed by an *in vivo* mouse RIRI model and an *in vitro* hypoxia/reoxygenation model, and tunicamycin-stimulated NRK-52E cells were used to explore the GLPP-mediated alleviation of ER stress. Experimental results showed that renal dysfunction and morphological damage were reduced in GLPP-treated group. The imbalance of redox status was reversed and production of ROS was reduced by GLPP. RIRI-induced mitochondrial- and ER stress-dependent apoptosis were dramatically inhibited in GLPP-treated group. Intriguingly, JNK activation in the kidney with RIRI or hypoxia/reoxygenation was inhibited by GLPP. These results suggest that the protective effect of GLPP against RIRI may be due to reducing oxidative stress, alleviating the mitochondrial and ER stress-dependent apoptosis caused by excessive ROS.

*Ganoderma lucidum* has been widely used as a traditional medicine in Asian countries to treat diseases, such as tumors^1^,^2^,^3^, liver disorders^4^, hypercholesterolemia^5^, obesity^6^ and cerebral ischemia reperfusion (IR)^7^. *Ganoderma lucidum* polysaccharide peptide (GLPP) was isolated from boiling water extract of the fruiting body of *Ganoderma lucidum* (Leyss ex Fr) Karst (*Gl*), followed by ethanol precipitation, dialysis and protein depletion using the Sevag method. This polysaccharides peptide has a molecular weight of approximately 5 × 10^5^ with a polysaccharide to peptide ratio of approximately 95%/5%. The polysaccharides consist of *D*-rhamnose, *D*-xylose, *D*-fructose, *D*-galactose, and *D*-glucose with molar ratios of 0.549:3.614:3.167:0.556:6.89 and linked together by β-glycosidic linkages^8^. GLPP is the major pharmacological constituent of *Ganoderma lucidum* and has diverse bioactivities^9^,^10^,^11^, among which, its antioxidant and radical-scavenging features suggest that GLPP may play a role in the pathophysiological mechanisms of renal ischemia reperfusion injury (RIRI).

RIRI inevitably occurs during surgery to treat occlusion of the renal arteries or the aorta and is a leading cause of perioperative acute kidney injury (AKI). AKI, characterized by an abrupt decrease in the glomerular filtration rate, is a common surgical complication that leads to unacceptably high mortality, chronic kidney disease (CKD), and end-stage renal disease^12^. RIRI involves a complex and interrelated sequence of events that result in the injury of renal cells and eventual cell death due to apoptosis and necrosis^13^. Although reperfusion is essential for the survival of ischemic tissue, reperfusion itself causes additional cell injury, which has been attributed to calcium overload, neutrophil infiltration and the generation of ROS^14^. Clinical and experimental studies have discovered that ROS play a vital role in tissue damage and cell apoptosis during IR, particularly during the process of reperfusion. ROS cause lipid peroxidation of biological membranes, disrupting structural integrity and energy production, especially in the proximal tubule segment highly susceptible to acute ischemia and hypoxia^15^,^16^.

During the process of RIRI, the mitochondria are the major sources and targets of ROS. Oxidative stress interferes with not only redox-dependent reactions but also with protein folding, ultimately resulting in protein misfolding in the endoplasmic reticulum (ER)^17^. Altered redox homeostasis in the ER is sufficient to cause ER stress, which in turn induces the production of ROS, both in the ER and in the mitochondria. Several studies have proven that ER stress and mitochondrial dysfunction are intimately linked to the pathogenesis of RIRI^18^. GLPP is able to reduce the accumulation of ROS that are closely associated with the pathophysiology of kidney failure and renal diseases^11^. Therefore, we proposed that GLPP may prevent and alleviate RIRI by restoring the balance of the oxidation/antioxidant system. In the current study, mouse RIRI model and a series of molecular pharmacology methods were used to investigate whether GLPP exerts a protective role against RIRI and its possible mechanisms involved were studied. The experimental results showed that GLPP could prevent RIRI, indicating that GLPP may be developed as a candidate drug for preventing RIRI.

## Results

### GLPP protected the kidney against RIRI

**Figure 1:**
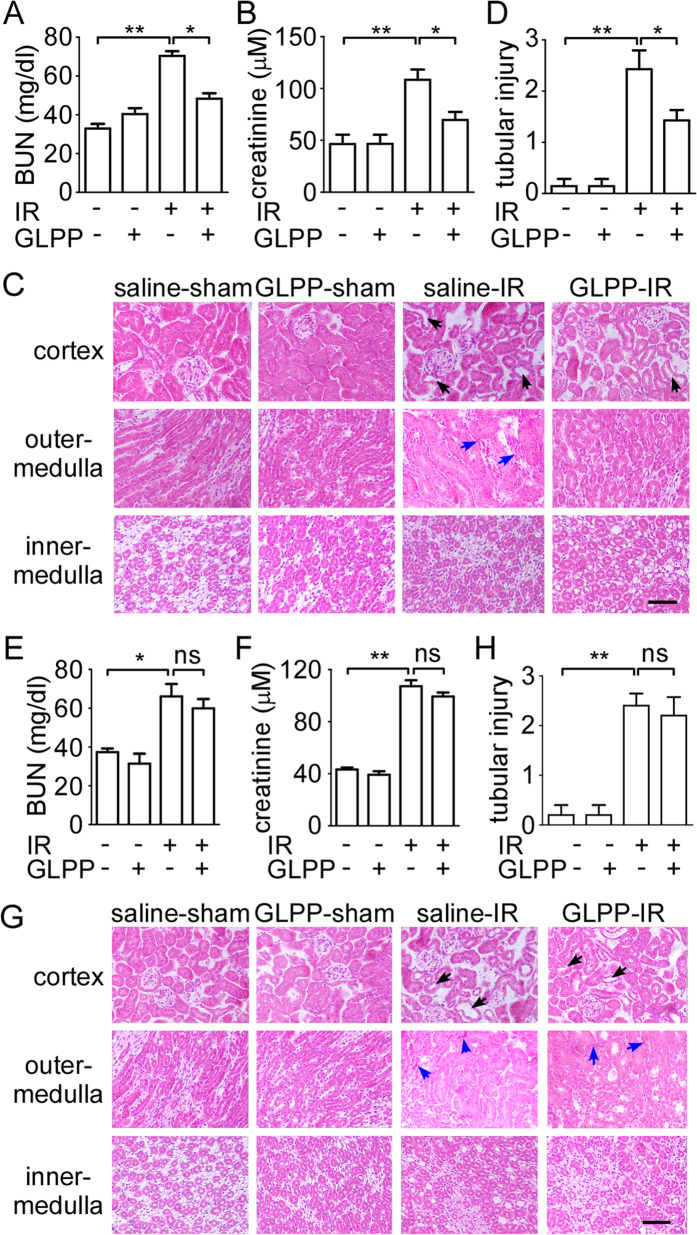
GLPP protected kidneys against RIRI. C57BL/6J male mice were intraperitoneally administered with vehicle or GLPP (100 mg/kg) daily for 7 days before surgery. Blood and kidney samples were collected for renal function tests and histological examination after reperfusion for 24 h. (**A**) BUN. (**B**) Blood creatinine. (**C**) Representative images of kidney tissue with H & E staining (magnification 400×). (**D**) Quantification of tubular injury. GLPP was intraperitoneally administered at the beginning of reperfusion to explore the therapeutic effect on IR. After reperfusion for 24 hours, blood and kidney samples were collected for analysis. (**E**) BUN in mice with GLPP post-treatment. (**F**) Blood creatinine in mice with GLPP post-treatment. (**G**) Representative images of kidney tissue with H & E staining in mice with GLPP post-treatment (magnification 400× ). (**H**) Quantification of tubular injury with GLPP post-treatment. Black arrows: tubular brush border loss and dilatation; Blue arrows: intertubular haemorrhage. Data are presented as the mean ± SEM (n = 8–10). ^*^P < 0.05, ^**^P < 0.01.

Renal function was assessed by the levels of blood urea nitrogen (BUN) and blood creatinine. Both parameters were significantly increased after renal IR compared with sham-operated mice. However, the administration of GLPP before ischemia and reperfusion resulted in improved renal function, as demonstrated by decreased BUN and creatinine levels (Fig. 1A,B).

Hematoxylin and eosin (H & E) staining was performed for the morphological analysis of renal tissues. Compared with sham-operated mice, proximal tubular damage including tubular brush border loss and dilatation and outer medulla injury including intertubular haemorrhage and congestion were found in the IR group. However, no significant damage was seen in inner medulla, which confirmed that the IR-induced renal injury was predominantly in proximal tubulars^16^. These changes were attenuated by GLPP pretreatment (Fig. 1C,D). Results above suggest that GLPP pretreatment exerts significant protective effect against RIRI.

We further explored whether postoperative administration of GLPP protected against RIRI. GLPP was intraperitoneally administered at the beginning of reperfusion. After 24 hours, blood and kidney samples were collected for analysis. GLPP did not significantly lower the IR-increased levels of BUN and blood creatinine (Fig. 1E,F). Additionally, post-treatment with GLPP did not observably reduce morphological changes caused by renal IR (Fig. 1G,H).

### GLPP modified renal oxidative stress and lipid peroxidation after IR

**Figure 2:**
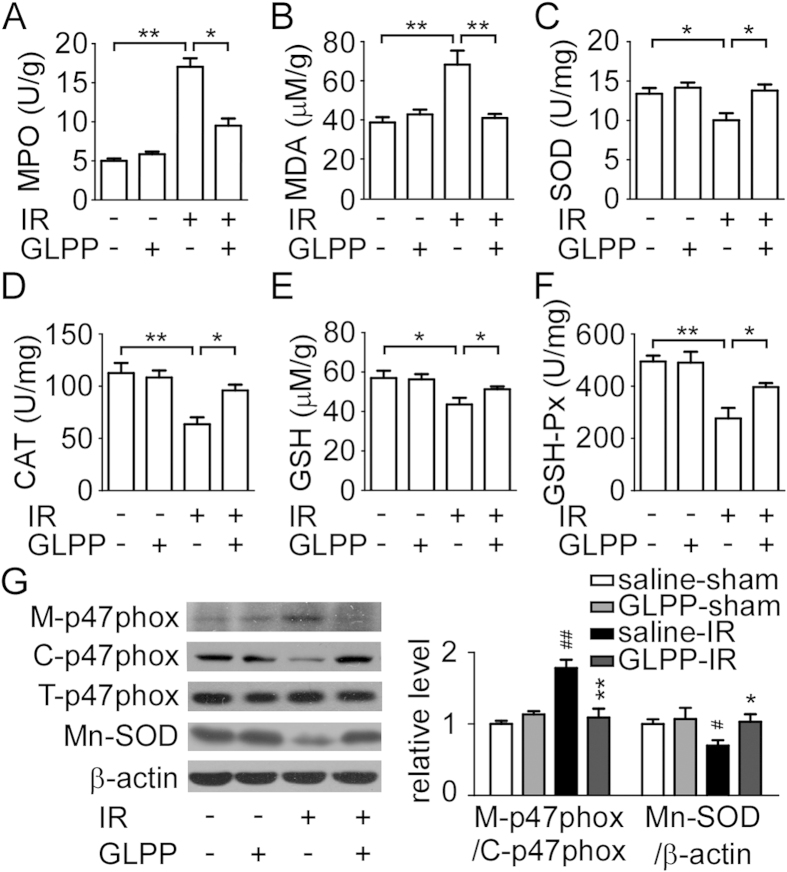
GLPP prevented renal oxidative stress and lipid peroxidation after IR. Kidney tissues were homogenized for evaluating the levels of different enzymes. (**A**) MPO activity in renal tissue. (**B**) MDA concentration in renal tissue. (**C**) SOD activity in renal tissue. (**D**) CAT activity in renal tissue. (**E**) GSH concentration in renal tissue. (**F**) GSH-Px activity in renal tissue. (**G**) Key enzymes involved in oxidative stress and protein levels. C-p47phox: Cytosol p47phox; M-p47phox: Membrane p47phox; T-p47phox: Total p47phox. Data are presented as the mean ± SEM (n = 8 − 10). ^*^P < 0.05, ^**^P < 0.01.

Oxidative stress plays a key role in the activation of mitochondrial and ER stress, which results in a series of abnormalities such as apoptosis, necrosis and other serious consequences. Myeloperoxidase (MPO), malondialdehyde (MDA), superoxide dismutase (SOD), catalase (CAT), reduced glutathione (GSH) and glutathione peroxidase (GSH-Px) were detected for evaluating the effect of GLPP on IR-mediated oxidative stress in the kidney. Compared with the sham group, RIRI significantly increased the levels of MPO and MDA and decreased the activities of SOD, CAT, GSH and GSH-Px while GLPP reversed these changes caused by IR (Fig. 2A–F). We then detected the expression of manganese superoxide dismutase (Mn-SOD), which is an important cellular antioxidant enzyme. As shown in Fig. 2G, IR significantly decreased the expression of Mn-SOD while GLPP increased its expression. Additionally, we isolated cell membrane and cytosol proteins separately for evaluating the changes of p47phox, a core regulatory subunit of NADPH oxidase, to promote NADPH oxidase-dependent production of ROS. It was found that IR stimulated the expression and translocation of p47phox to the membrane while GLPP inhibited its translocation, suggesting that the beneficial effect of GLPP may be partially attributed to alleviating the NADPH oxidase-dependent production of ROS.

### GLPP inhibited IR-induced apoptosis by reducing mitochondrial and ER stress

**Figure 3:**
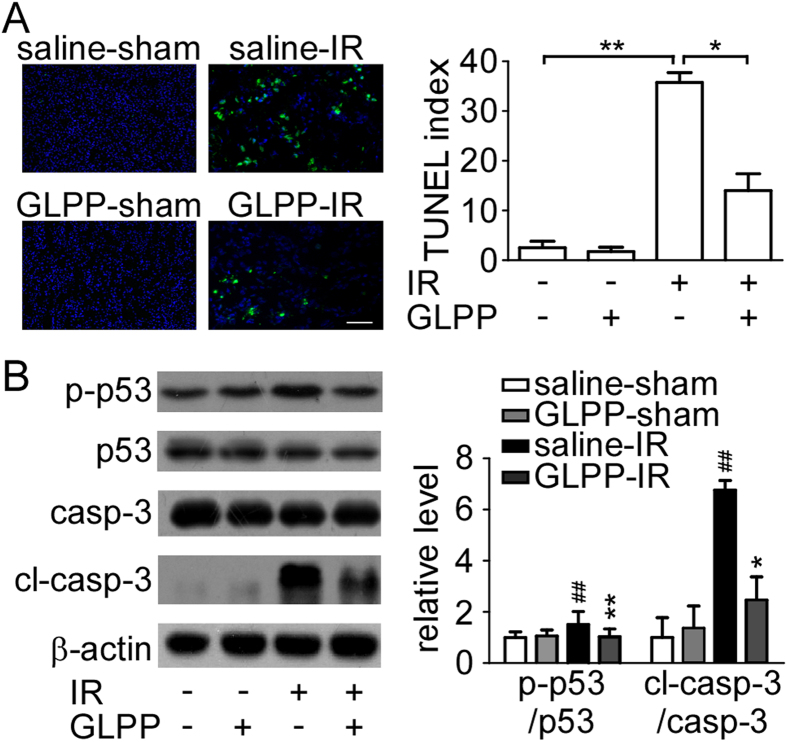
GLPP reduced cell apoptosis in the kidney after IR. (**A**) TUNEL staining (green fluorescence, magnification 200×) (left) and TUNEL positive index (right). (**B**) Western blots (left) and quantifications (right). The data were normalized by the intensity of β-actin and related to the value of the sham. Means ± SEM (n = 3). ^#^P < 0.05, ^##^P < 0.01 *vs.* sham; ^*^P < 0.05, ^**^P < 0.01 *vs.* saline-treated IR group.

Impaired redox status results in accumulation of ROS, thus activates mitochondrial and ER stress. When homeostasis is disrupted and adaptive responses fail to compensate for the stress, apoptosis is triggered. A TUNEL assay was used to evaluate apoptosis in renal tissues induced by IR. More apoptotic cells appeared in kidneys subjected to IR than in the sham-operated kidneys. GLPP reduced IR-induced TUNEL-positive cells by 21.75%, which suggests that GLPP protects kidneys from renal tubular apoptosis (Fig. 3A). Results were confirmed by Western blot analysis, demonstrated as decreased ratios of p-p53/p53 and cleaved caspase-3/caspase-3 in the IR GLPP-treated group (Fig. 3B).

**Figure 4:**
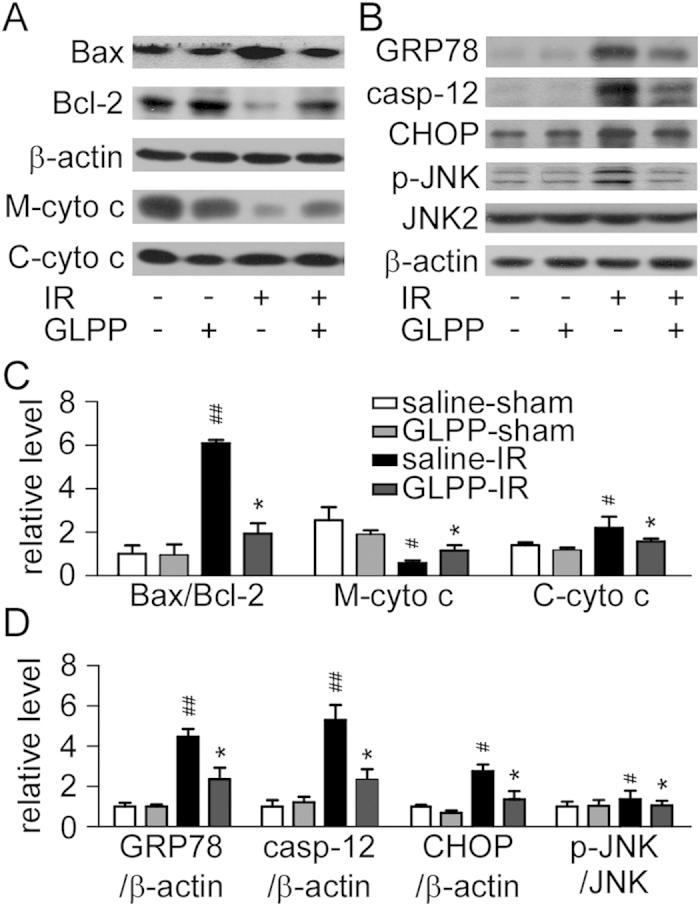
GLPP attenuated mitochondrial and ER stress after IR. (**A**) Western blots of mitochondrial function-related proteins. M-cyto c: Mitochondrial cytochrome c; C-cyto c: Cytosolic cytochrome c. (**B**) Western blots of ER stress-related proteins. (**C**) Quantification of mitochondrial function-related protein. (**D**) Quantification of ER stress-related protein.

We then analyzed the ratio of Bax/Bcl-2 that reflects mitochondrial-related apoptosis. GLPP reduced the ratio of Bax/Bcl-2 caused by IR, which demonstrated that GLPP restored mitochondrial function. Correspondingly, we isolated mitochondrial fractions from IR kidneys and detected the expression of cytochrome c in both the mitochondrial and cytosolic fractions. The results showed that IR caused more cytochrome c release from mitochondria to the cytosol while GLPP reduced the ratio of Bax/Bcl-2 and inhibited the release of cytochrome c (Fig. 4A). These results indicate that GLPP inhibits the mitochondria-dependent apoptosis induced by IR.

The expression of 78 kDa glucose-regulated protein (GRP78), CCAAT/enhancer-binding protein (C/EBP)-homologous protein (CHOP) and caspase-12 was increased after IR, which suggested that the kidneys had undergone serious ER stress. Additionally, the phosphorylation of JNK increased in the IR group, which played a pivotal role in ER stress-induced apoptosis. GLPP reduced the expression of these ER stress biomarkers and inhibited the activation of JNK (Fig. 4B), indicating that GLPP inhibits IR-induced apoptosis, presumably by alleviating ER stress.

### GLPP increased cell viability and reduced cell oxidative stress induced by hypoxia/reoxygenation (H/R)

**Figure 5:**
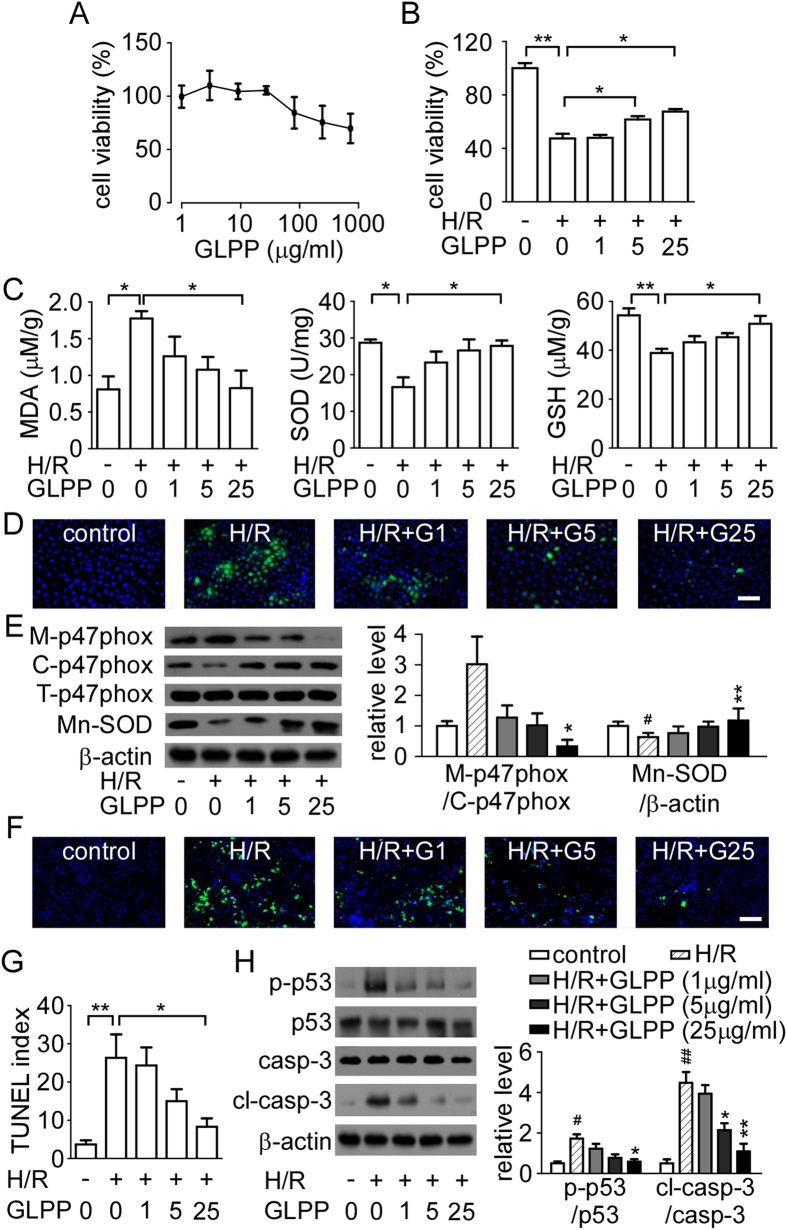
GLPP reduced cell oxidative stress and inhibited cell apoptosis. (**A**) Cytotoxicity of GLPP on NRK-52E cell. (**B**) Effect of GLPP on NRK-52E cell viability under H/R conditions. (**C**) Biomarkers of oxidative stress in NRK-52E cells. (**D**) ROS production (magnification 200×). (**E**) Key enzymes expression involved in oxidative stress. (**F**) TUNEL staining (magnification 200×). (**G**) Quantification of TUNEL positive index. (**H**) Apoptotic proteins levels. Means ± SEM (n = 3). ^#^P < 0.05, ^##^P < 0.01 *vs.* control group; ^*^P < 0.05, ^**^P < 0.01 *vs.* H/R-treated group.

A CCK-8 assay was used to assess the cytotoxicity of GLPP. At concentrations from 1.1 μg/ml to 810 μg/ml, GLPP showed no obvious cytotoxicity on NRK-52E cells (Fig. 5A). Hypoxia for 12 h followed by reoxygenation for 1 h significantly decreased the cell viability compared to control cells. Pretreatment with GLPP improved cell viability in a dose-dependent manner (Fig. 5B).

We then studied the effect of GLPP against cellular oxidative stress. The H/R significantly reduced the activities of SOD and GSH, which was associated with a reciprocal increase in MDA level and ROS production (Fig. 5C,D). The translocation of p47phox to membrane was inhibited and the expression of Mn-SOD was increased by GLPP in a dose dependent manner (Fig. 5E), indicating that GLPP has a protective role against H/R by inhibiting the activation of NADPH oxidase and increasing antioxidant activity, which finally reduces the accumulation of ROS and alleviates the oxidative stress.

### GLPP inhibited cell apoptosis induced by H/R

NRK-52E cell apoptosis was analyzed by TUNEL assays and Western blot assays. More apoptotic cells appeared in the H/R group compared with the control group while GLPP significantly reduced H/R-increased apoptotic cells (Fig. 5F), which was in accordance with the *in vivo* results. The protein levels of p-p53, p53, cleaved caspase-3 and caspase-3 were further tested (Fig. 5H). In line with the mouse model, we found that H/R increased cell apoptosis by means of up-regulating of the ratios of p-p53/p53 and cleaving capsase-3/caspase-3. However, pretreatment with GLPP reversed these ratios in a dose-dependent manner, which confirmed the results of the TUNEL analysis. All these data suggest that GLPP has a protective effect against H/R-induced apoptosis in renal tubular cells.

### GLPP attenuated H/R-induced mitochondrial dysfunction

**Figure 6:**
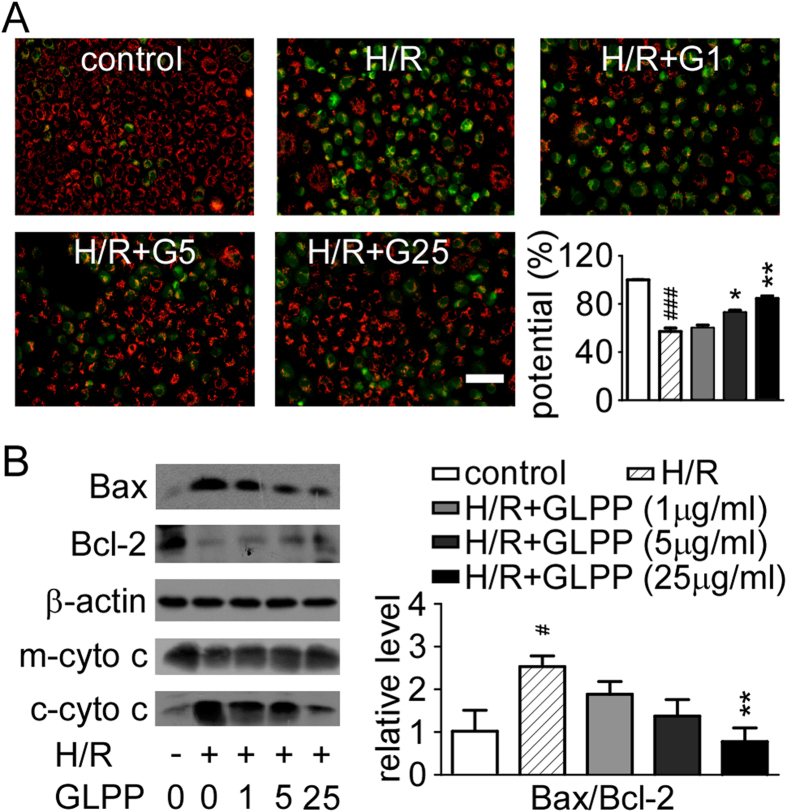
GLPP attenuated H/R-induced mitochondrial dysfunction. (**A**) ΔΨm of mitochondria. Bar diagram shows the ratio of red fluorescence to green fluorescence. (**B**) Western blots (left) and quantifications (right).

To determine whether H/R affected mitochondrial function, NRK-52E cells were analyzed by fluorescent, lipophilic and JC-1 (cationic probe) staining. We found that H/R resulted in significant dissipation of mitochondrial ΔΨm, indicated by increased green fluorescence. GLPP-pretreated cells exhibited attenuated ΔΨm dissipation caused by H/R (Fig. 6A), which indicates that GLPP pretreatment diminishes H/R-induced mitochondrial dysfunction. Increased Bax expression and decreased Bcl-2 expression were found in the H/R group while GLPP reversed these expression changes. Furthermore, H/R resulted in cytochrome c releasing from the mitochondria into the cytosol, which was suppressed by GLPP in a dose-dependent manner (Fig. 6B).

### GLPP reduced ER stress-dependent apoptosis

**Figure 7:**
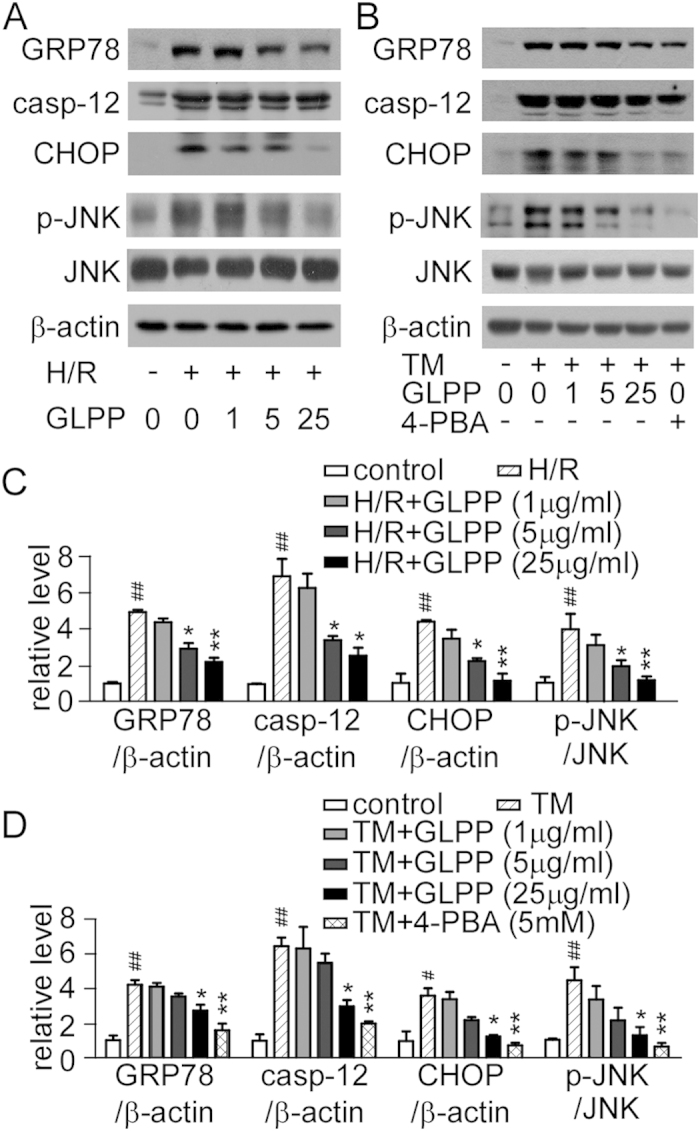
GLPP reduced H/R-induced ER stress. (**A**) NRK-52E cells were exposed to hypoxia for 12 h and reoxygenation for 1 h. Representative Western blots of the ER stress biomarkers were as shown. (**B**) NRK-52E cells were treated for 24 h with TM (2 μg/ml) or an equal volume of DMSO as the vehicle control. GLPP was given for 12 h before the TM, whereas 4-PBA (5 mM) was administered at the onset of the TM for 24 h, then the ER stress biomarkers were detected. (**C**) Protein levels in experiments shown in (**A**). (**D**) Protein levels in experiments shown in (**B**).

To explore the influence of GLPP on H/R-induced ER stress, we then tested the changes in GRP78, caspase-12 and CHOP. H/R increased the expression of these proteins, whereas GLPP dramatically reversed these changes (Fig. 7A). Afterwards, tunicamycin (TM), an ER stress inducer, was used to stimulate NRK-52E cells and to create a special ER stress model. We found that TM significantly increased the expression of GRP78, caspase-12 and CHOP, suggesting that NRK-52E cells underwent serious ER stress. Interestingly, both H/R and TM increased the phosphorylation of JNK, while 4-PBA, a specific inhibitor of ER stress, or GLPP significantly inhibited the expression of p-JNK and ER stress markers (Fig. 7B), demonstrating that GLPP relieves ER stress-induced apoptosis at least partially through the JNK signaling pathway (Fig. 7B).

## Discussion

The aim of this study was to determine whether GLPP could protect kidneys against RIRI and to elucidate the related mechanisms. RIRI is a common cause of AKI in patients during renal transplantation or with recanalization after occlusion of renal blood flow. Mice RIRI model is generally used to study the mechanisms in which AKI occurs and to evaluate potential anti-AKI activity of active compounds. In the current study, GLPP restored the balance of oxidative stress induced by IR, indicating a protective effect of GLPP against IR, likely related to improvement in the endogenous antioxidant system.

MDA is an index of oxidative stress and also a prominent product of lipid peroxidation^19^. SOD is an indicator of anti-oxidative capacity, involved in reversing the pathological changes in oxidative injury. It is well accepted that SOD, CAT, GSH and GSH-Px play important roles in the endogenous defense system against oxygen free radicals^20^,^21^. Generally, increased MDA and decreased SOD, CAT, GSH, GSH-Px in kidney tissue after IR has been documented^22^,^23^. In the current study, administering GLPP before IR or treatment with GLPP before H/R decreased renal MDA and increased endogenous antioxidant enzymes. We also found that the increased ROS production and decreased Mn-SOD expression caused by IR or H/R were reversed by GLPP. Interestingly, it was found that the IR or H/R induced activation of NADPH oxidase was significantly inhibited by GLPP. All these results indicate that GLPP may reduce the NADPH oxidase-dependent production of ROS and increase ROS elimination to normalize the imbalance between the anti-oxidative and oxidative status after IR.

Accumulated ROS may activate mitochondrial stress pathways to cause mitochondrial injury. Mitochondria are the main sources of ROS in the process of reperfusion, and contribute critically to the pathogenesis of IR by activating the signaling pathways of cell injury and apoptosis. High ROS activity indicates depolarization of the mitochondrial membrane, which increases the expression of the pro-apoptotic protein Bax on the outer mitochondrial membrane^24^. Bax is a membrane protein of the Bcl-2 family that participates in regulating mitochondrial membrane permeabilization and the mitochondrial-dependent pathway of apoptosis^25^,^26^. In renal cells, IR or H/R increases the expression of Bax and decreases the expression of Bcl-2. Altered ratio of Bax/Bcl-2 promotes the release of cytochrome c. Accumulated cytochrome c in the cytosol activates caspase-9, which is responsible for the apoptotic initiation process^27^. The activated caspase-9 then proteolytically cleaves and activates executioners such as caspase-3, which eventually trigger cell apoptosis^28^. In our study, H/R resulted in significant dissipation of mitochondrial ΔΨm and increased ratios of Bax/Bcl-2 and cleaved caspase-3/caspase-3. In addition, more cytochrome c was released from the mitochondria to the cytosol, which indicated that the cells had undergone apoptosis via a mitochondria-dependent pathway.

Previous studies have proven that the *G. lucidum* peptides play substantial protective roles in rat liver tissue homogenates and mitochondrial membrane peroxidation systems through their antioxidant, metal-chelating, and free radical-scavenging activities^29^. GLPP may also protect the mitochondria from tert-butylhydroperoxide (t-BOOH)-caused injury due to its antioxidative capacity^11^. Our data showed that GLPP treatment restored the balance of oxidative stress, ameliorated mitochondrial function and reduced cell apoptosis in IR. Basing on these findings, we propose that GLPP alleviates oxidative stress by reducing ROS production and accumulation, thus reducing mitochondrial stress-dependent apoptosis.

ER stress is one of the various mechanisms contributing to cellular damage and apoptosis^30^,^31^. Previous studies strongly suggest that ER stress plays a key role in the pathogenesis of several renal cell injury and kidney diseases, including renal tubular epithelial cell apoptosis^32^, podocyte injury in diabetic nephropathy^33^ and IR-induced AKI^34^. Excessive ER stress triggered by ischemia or H/R usually results in the up-regulation of the ER stress response protein GRP78^35^. GRP78 is an ER chaperone, a key unfolded protein response (UPR) with multiple roles in protein processing and cellular protection and is a marker of ER stress^36^. CHOP is considered as an ER stress-associated pro-apoptotic protein and the target of UPR signaling pathways and pro-apoptosis during the ER stress response in AKI^37^,^38^.

Caspase-12, expressed ubiquitously and constitutively, is exclusively located in the ER^39^. Unlike other caspases, caspase-12 is specifically activated by insult-induced ER stress-dependent apoptosis^40^,^41^. Caspase-12 and p-JNK were up-regulated in the IR-exposed kidney^18^ and over-expressed in the diabetic kidney^42^. Activated JNK translocates to mitochondrial membrane, where it is decisive for cytochrome c release. Studies have shown that JNK is involved in ER stress-induced apoptosis in lung epithelial cells^43^ and HeLa cells^44^. Additionally, the concomitant occurrence of both ER stress markers and JNK activation indicate that JNK activation occurs downstream of ER stress^45^ and that the ER stress-JNK pathway plays an important role in mediating insulin resistance and apoptosis^46^,^47^.

In our study, the Western blot analysis revealed that the expression of GRP78, CHOP and caspase-12 elevated notably after IR and H/R, indicating that ER stress participated in IR or H/R induced apoptosis. However, GLPP pretreatment reduced these parameters. To further confirm that GLPP protects against AKI by ameliorating ER stress, we built an ER stress model *in vitro* by incubating NRK-52E cells with TM for 24 h. Intriguingly, GLPP alleviated TM-induced ER stress in a dose-dependent manner, which firstly proved that GLPP could directly inhibited the ER stress. The activation of JNK was involved in TM-induced ER stress, while 4-PBA or GLPP inhibited its activation, suggesting that the JNK signaling pathway is involved in ER stress-induced apoptosis, and the renal-protective effect of GLPP may be attributed to the alleviation of ER stress-dependent apoptosis.

**Figure 8:**
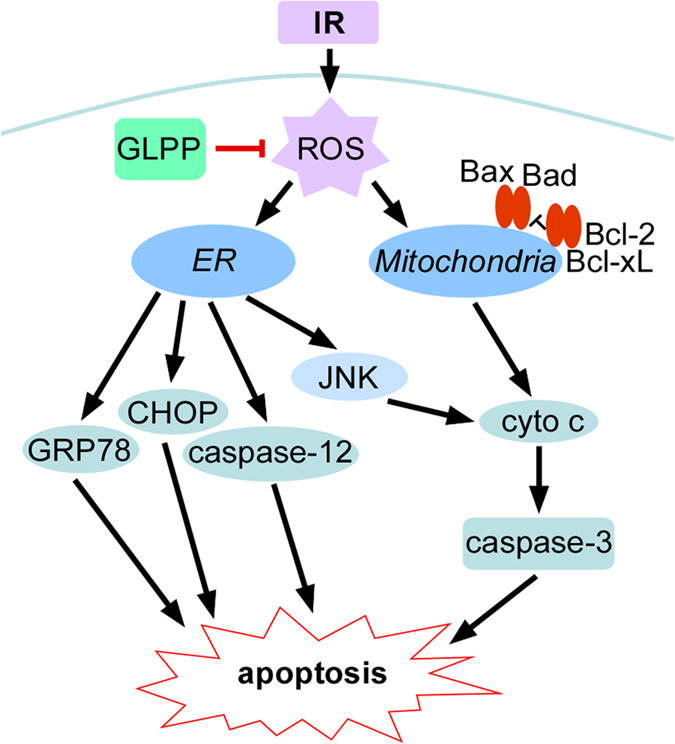
Schematic diagram of the signal pathways involved in IR-induced apoptosis. Please see the text for explanations.

Based on our experimental data, we suggest a mechanism in which GLPP protects the kidneys from RIRI (Fig. 8). RIRI results in the generation and accumulation of ROS in renal tissue, which surpasses the scavenging capacity of endogenous antioxidases. The excessive ROS activate both mitochondrial stress and ER stress pathways. When the mitochondria are impaired, the increased Bax and decreased Bcl-2 alters the ratio of Bax to Bcl-2 in the outer membrane of mitochondria and promotes the release of cytochrome c. Released cytochrome c activates the apoptotic effector protein, caspase-3, which eventually induces mitochondrial-dependent cell apoptosis. At the same time, accumulated ROS also activate ER stress, showing as the increased expression of GRP78, activated caspase-12 and CHOP, which ultimately triggers the onset of ER stress-dependent apoptosis. ER stress may also activate the JNK signaling pathway, which could enhance the release of cytochrome c and further contribute to cell apoptosis.

*Ganoderma lucidum* has been reported to have wide effects in suppressing inflammation^48^ and increases the activity of SOD in cerebral IR^7^. MPO is a characteristic constituent of neutrophil granules and represents ROS-related inflammation. In our study, GLPP treatment reduced IR-induced MPO activity in kidney, suggesting that a potentially harmful effect of MPO in immune-mediated inflammation might be alleviated by GLPP. It has been reported that immune response and inflammation in RIRI^49^,^50^,^51^ and GLPP exerts beneficial effect on injury of macrophages by inhibiting the foam cell formation and necrosis^21^. The effect of GLPP on immune response and inflammation in RIRI and related mechanism need to be further explored.

Previous studies have showed effects of *Ganoderma lucidum* and its extracts on renal injury. The hot water extract of *Ganoderma lucidum* dose-dependently reduced mouse kidney lipid peroxidation induced by 95% ethanol^52^. Other studies indicated that *Ganoderma* extract significantly reduced oxidative damage and apoptosis in human proximal tubular epithelial cells induced by human serum albumin^53^, while Lingzhiols, extracted from *Ganoderma lucidum*, could inhibit TGF-β/Smads signaling pathways^54^. Moreover, a novel proteoglycan from *Ganoderma lucidum* fruiting bodies conferred significant amelioration on renal function and morphologic injuries in diabetic nephropathy mice^55^. These studies suggest that *Ganoderma lucidum* and its extracts have wide renal protective activities and a good application prospect in different kidney diseases.

In summary, the current study verifies, for the first time, that GLPP has beneficial effects on IR caused AKI. The protective effect of GLPP against RIRI may be attributed to the inhibition of NADPH oxidase-dependent production of ROS and the increase of free radical-scavenging capacity for the balance of the oxidation/antioxidant system, improving mitochondrial dysfunction and ER stress-dependent apoptosis, which subsequently alleviates AKI caused by IR. Our study suggests that GLPP may be developed as a candidate drug for preventing AKI.

## Materials and Methods

### GLPP

GLPP is a hazel-colored, water-soluble powder, kindly provided by Fuzhou Institute of Green Valley Bio-Pharm Technology. The compound’s average molecular weight is approximately 520 kDa, as determined by high-performance steric exclusion chromatography analysis. In the experiments, GLPP was dissolved in physiological saline for animal treatment and in PBS for cell incubation.

### Ethics statement

All procedures in this study were carried out in strict accordance with the recommendations in the Guide for the Care and Use of Laboratory Animals of China Association for Laboratory Animal Science. All animal care protocols were approved by the Animal Care Committee of Peking University Health Science Center. All sacrifices were performed under pentobarbitone anesthesia, and every effort was made to minimize animal suffering.

### Acute renal injury mouse model

Male C57BL/6J mice (8 weeks-10 weeks old) weighing 20 g–22 g were purchased from the Animal Center of Peking University Health Science Center. The animals were housed with a 12 h/12 h light/dark cycle, and food and water were available *ad libitum*. The mice were divided randomly into four groups: the sham-operated group; the sham-operated GLPP-treated group; the IR group; and the IR GLPP-treated group. For the warm renal IR, the mice were anesthetized by intraperitoneal injections of sodium pentobarbital (80 mg/kg). Then, the right renal pedicle was identified and occluded with a small vascular clamp for 35 min while the left kidney was removed. For reperfusion, the clamp was released and the kidney was monitored by a change in the color of the kidney to confirm blood reflow before suturing the incision. Sham-operated animals underwent an identical operation without renal pedicle clamping. In order to analyze the prevention efficacy of GLPP against IR injury, 100 mg/kg/day of GLPP was intraperitoneally injected for 7 days before the procedure until sacrifice. For evaluating the therapeutic effect of GLPP on RIRI, GLPP was given by intraperitoneal injection at the onset of reperfusion. After reperfusion for 24 h, blood and kidney samples were collected for further study.

### Blood BUN and creatinine measurement

After reperfusion for 24 h, blood samples were collected to determine the levels of blood BUN and creatinine. BUN was measured using a quantitative colorimetric urea determination kit (QuantiChrom Urea Assay Kit-DIUR-500). Blood creatinine concentrations were measured with commercial kits (NJJC Bio, Nanjing, China), according to the manufacturer’s instructions.

### H & E staining

Kidneys were fixed with 4% formaldehyde for subsequent paraffin embedding and sectioning. The sections were stained with H & E for morphological analysis. Tissue damage was assessed using a tubular damage score, as previously described^56^. Briefly, injury was scored in a blinded manner according to the percentage of damage included loss of brush border, tubular dilation and intertubular haemorrhage: 0, no damage; 1, < 25%; 2, 25 ~ 50%; 3, 50 ~ 75%; 4, > 75%. Representative fields were captured.

### Measurement of biomarkers of oxidative stress

Homogenized renal samples were tested for MPO, MDA, SOD, CAT, GSH and GSH-Px activities using specific assay kits (NJJC Bio, Nanjing, China) according to the manufacturer’s instructions.

### Cell culture

The NRK-52E cells (rat renal proximal tubule epithelial cells) were purchased from the Cell Resource Center of Shanghai Institutes for Biological Sciences, Chinese Academy of Sciences (Shanghai, China). Briefly, the NRK-52E cells were cultured in DMEM containing 10% fetal bovine serum (FBS; Gibco), 2 mM glutamine, 100 U/ml penicillin and 100 μg/ml streptomycin, in a humidified atmosphere with 5% CO_2_ at 37 °C

### Cell H/R

The NRK-52E cell H/R model was designed to mimic renal cell I/R injury *in vitro*. Before the procedure, the NRK-52E cells were cultured to 70% to 80% confluence, and then they were serum deprived for 24 h. GLPP, at a concentration of 1 μg/ml, 5 μg/ml or 25 μg/ml, was added to the cell cultures 12 h before hypoxia. In all the H/R processes, the cells were incubated in starving low-glucose DMEM under low-oxygen (1% O_2_) for 12 h in a humidified hypoxia incubator (Thermo Fisher Scientific, USA). The cells were then exposed to normal oxygen (95% air + 5% CO_2_) for 1 h. After the completion of the procedure, the supernatant and cells were collected separately for further analysis. Control groups were cultured in normal conditions, coincident with the duration of H/R injury, and the supernatant and cells were harvested separately for further analysis.

### Cytotoxicity and viability assay

The CCK-8 assay kit (Dojindo) was used for testing cytotoxicity *in vitro*. NRK-52E cells were planted in 96-well plates at a density of 5000 cells/well. GLPP was co-incubated with NRK-52E cells for 24 h. Then, the CCK-8 solution, at a 1/10 dilution with 10% FBS DMEM, was added to each well, and the cells were incubated for 3 h at 37 °C. Absorbance at 450 nm was measured with a microplate reader (Biotek, MQX200). To analyze the effect of GLPP on cell viability in NRK-52E affected by H/R, the cells were pretreated with GLPP for 12 h, and the H/R protocol was performed. Cell viability was calculated as follows:




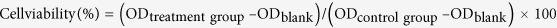




### Detection of intracellular ROS production and biomarkers of oxidative stress

Cells planted in 96-well plates were subjected to H/R protocol. After reoxygenation, cells were washed twice with PBS and incubated in the presence of 10 μM 2′-7′-Dichlorodihydroflurescein diacetate (DCFH-DA, Sigma) in serum free DMEM for 30 min at 37 °C. DCFH-DA was de-esterified intracellularly and turned into highly fluorescent 2′-7′-dichlorofluorescein upon oxidation by cellular esterases. Levels of intracellular oxidative stress were reflected by DCF fluorescence intensity (excitation wavelength 485 nm, fluorescence wavelength 530 nm).

Cells were collected for detection of MDA, SOD and GSH activities using specific assay kits (NJJC Bio, Nanjing, China) according to the manufacturer’s instructions.

### Terminal deoxynucleotidyl transferase-mediated 2’-deoxyuridine 5’-triphosphate nick-end labeling assay (TUNEL)

Kidney tissues embedded in Tissue-Tek Optimal Cutting Temperature (OCT) compound or cells planted in 96-well plates were prepared for TUNEL analysis using the *in situ* Cell Death Detection kit (Roche Applied Science) according to the manufacturer’s instructions. Cells with positive nuclear staining with DNA breakage were identified and counted by fluorescence microscopy. The apoptotic index was defined as ((number of apoptotic cells/total number of nucleated cells) ×100).

### Mitochondrial membrane potential (∆Ψm) measurement

∆Ψm of NRK-52E cells was measured by a fluorescent, lipophilic and cationic probe, JC-1 (Beyotime), according to the manufacturer’s instructions. NRK-52E cells were planted in 96-well plates at a density of 5000 cells/well, and the H/R protocol was performed as above. Then, the cells were incubated with JC-1 staining solution for 20 min at 37 °C. Fluorescence was detected with a Fluostar Optima microplate reader (BMG Technologies). The wavelengths of excitation and emission were 490 nm and 535 nm for detection of monomeric form of JC-1. 525 nm and 590 nm were used to detect aggregation of JC-1. The ratio of ‘red’ to ‘green’ fluorescence represented ∆Ψm of NRK-52E cells.

### ER stress assessment

To assay ER stress, cells were cultured to 70% to 80% confluence and then pretreated with GLPP for 12 h. Subsequently, TM (2 μg/ml) or 4-PBA (5 mM) (Sigma) was incubated with the cells in DMEM containing 10% FBS for 24 h. After the completion of the experiments, the cells were collected for Western blot analysis.

### Western blot analysis

Tissues or cells were homogenized in RIPA lysis buffer containing a protease inhibitor cocktail (Roche). Membrane protein, cytosolic protein and mitochondrial protein were isolated using the Membrane/Cytosol or Mitochondria/Cytosol Protein Fractionation Kit according to the manufacturer’s protocol (Beyotime Inst Biotech). Identical amounts of protein samples were separated by SDS-PAGE, blotted onto a PVDF membrane, and incubated with antibodies against p47phox (Bioworld), Mn-SOD (ABclonal), Bax, Bcl-2, caspase-12, CHOP, p-JNK, β-actin (Santa Cruz), p53, caspase-3, cleaved caspase-3, GRP78 (Cell Signaling Technology), JNK (ABclonal), p-p53, or cytochrome-c (Abcam) at 4 °C overnight. Then, goat anti-rabbit IgG or goat anti-mouse IgG (Santa Cruz) were added, and the blots were developed with an ECL plus kit (Amersham Biosciences). The images were scanned with an Epson scanning system, and the data were analyzed with Quantity-one software. The data are expressed as the values relative to the sham or control value.

### Statistical analyses

All results are represented as the mean ± SEM. Data involving only two groups was analyzed using a two-tailed student *t*-test assuming unequal variances. When more than two experimental groups were compared, the data was analyzed using the Tukey-Kramer test with Prism 5.0 software to compare data between individual experimental groups. A p-value of <0.05 was considered to be statistically significant for all tests.

## Additional Information

**How to cite this article**: Zhong, D. *et al.* *Ganoderma lucidum* polysaccharide peptide prevents renal ischemia reperfusion injury via counteracting oxidative stress. *Sci. Rep.* **5**, 16910; doi: 10.1038/srep16910 (2015).
